# Disrupted Brain Connectivity Networks in Aphasia Revealed by Resting-State fMRI

**DOI:** 10.3389/fnagi.2021.666301

**Published:** 2021-10-20

**Authors:** Xiaoyun Chen, Liting Chen, Senning Zheng, Hong Wang, Yanhong Dai, Zhuoming Chen, Ruiwang Huang

**Affiliations:** ^1^Department of Rehabilitation, The First Affiliated Hospital of Jinan University, Guangzhou, China; ^2^Medical Imaging Center, First Affiliated Hospital of Jinan University, Guangzhou, China; ^3^Key Laboratory of Mental Health and Cognitive Science of Guangdong, Center for the Study of Applied Psychology and MRI Center, School of Psychology, Institute of Brain Research and Rehabilitation (IBRR), South China Normal University, Guangzhou, China

**Keywords:** aphasia, cognitive impairment, topological properties, functional MRI, stroke

## Abstract

Aphasia is characterized by the disability of spontaneous conversation, listening, understanding, retelling, naming, reading, or writing. However, the neural mechanisms of language damage after stroke are still under discussion. This study aimed to investigate the global and nodal characterization of the functional networks in patients with aphasic stroke based on resting-state functional MRI (fMRI). Twenty-four right-handed patients with aphasia after stroke and 19 healthy controls (HC) underwent a 3-TfMRI scan. A whole-brain large-scale functional connectivity network was then constructed based on Power's atlas of 264 functional regions of interest, and the global and nodal topological properties of these networks were analyzed using graph theory approaches. The results showed that patients with aphasia had decreased in small-worldness (sigma), normalized clustering coefficient (gamma), and local efficiency (*E*_loc_) values. Furthermore, *E*_loc_ was positively correlated with language ability, retelling, naming, and listening comprehension in patients with aphasia. Patients with aphasia also had decreased nodal degree and decreased nodal efficiency in the left postcentral gyrus, central opercular cortex, and insular cortex. Our results suggest that the global and local topology attributes were altered by injury in patients with aphasic stroke. We argue that the local efficiency of brain networks might be used as a potential indicator of basic speech function in patients with aphasia.

## Introduction

Aphasia, disruption of cognitive processes underlying language, manifests as impairments in spontaneous conversation, auditory comprehension, retelling, naming, reading, or writing. Notably, aphasia is closely associated with higher morbidity, mortality, and societal costs. Stroke is the most common cause of aphasia (especially in occlusion within the middle cerebral artery territory), and about 20–40% of strokes leading to acute aphasia (Engelter et al., 2006).

For a long time, one of the important models to help us understand the pathological mechanism of aphasia is the Wernicke-Lichtheim model (Lichteim, 1885; Wernicke, 1984), which includes Broca's and Wernicke's areas, and white matter fiber bundles (arcuate bundle) connecting these two brain regions. This model asserts that each of these brain regions has specialized functions, that neuroanatomical pathways support communication between them, and that this can be used to understand the interpretation of language or early clinical symptoms. It is particularly useful in the neurobiological study; however, with increased understanding of and research into the structure and function of the brain, the limitations of this early classical model of language have become increasingly prominent.

With the development of research technology, a new language model, the dual-stream model (Hickok and Poeppel, 2004, 2007) has gradually emerged. This model pays more attention to the connection between the cortex, and it mainly describes two large-scale processing streams: the ventral and dorsal streams. The ventral flow supports auditory-to-semantic information processing and listening comprehension. This area is mainly located in the lateral temporal lobe, extending into the posterior-inferior frontal gyrus pars orbitalis *via* the uncinate fasciculus. The dorsal stream, which processes information from hearing to pronunciation, is located at the junction between the left frontal language area and the temporal parietal area. It mainly provides auditory and proprioceptive feedback, which is important for fluent speech. The major brain regions involved are frontoparietal regions, including the pars opercularis, pars triangularis, pre- and postcentral regions, and portions of the parietal lobe. Although the dorsal and nerve streams provide the crucial organizational framework for human communication, they are not equally important for language processing and speech. For instance, damage to the cerebral cortex, angular gyrus, or posterior cortex is generally more harmful to speech and language processing than damage to other regions based on region-wise lesion-symptom mapping (Fridriksson et al., 2018). Additionally, there are symptomatic differences between injuries to brain areas associated with producing increasingly complex word combinations and areas associated with producing syntactically accurate connected speech. Inability to produce complicated language structure is associated with damage to the posterior temporal cortex and the angular gyrus of the inferior parietal lobe. In contrast, defects in syntactic accuracy are associated with damage to the left inferior frontal gyrus, insular, postcentral gyrus, precentral gyrus, and supramarginal gyrus of the inferior parietal lobe (Fridriksson et al., 2018).

The graph theory analysis method is centrally important to understanding the structure and function of networked brain systems, including their architecture, development, and evolution. Based on the strong influence of the dual stream model, we used a graph theory analysis method to explore the local and global topological properties of brain functional networks in patients with aphasia and explore the relationships between these indicators and aphasia symptoms. An increasing number of studies have used graph theory in conjunction with functional connectivity (FC) to investigate the topological properties of brain functional networks and the effects of focal damage on functional brain networks (Baliki et al., 2018; Tao and Rapp, 2019; Johnson et al., 2020). Tao and Rapp (2019) investigated the properties of functional networks in aphasia, and their investigation revealed that more modular networks with higher local integration resulted in a greater response to treatment and were associated with lower deficit severity; both global network modularity and local integration increased after treatment, especially within intact ventral occipital-temporal regions of the spelling network. Johnson et al. (2020) also used graph analysis to examine a network of regions linked to semantic processing in aphasia who went on to receive naming treatment. They found that aphasia with higher pre-treatment graph metrics responded better to treatment than those with lower network measures. They accordingly suggested that higher global efficiency and strength in the semantic network might be favorable prognostic indicators for improvement from semantic naming treatment. In addition, Baliki et al. (2018) used graph theory analysis to investigate the influence of baseline anatomical and functional brain properties in response to intensive comprehensive aphasia treatment based on resting-state functional MRI (fMRI). They found that baseline resting-state FC (rsFC) properties in aphasia were strongly associated with treatment outcomes, and that large-scale properties, such as global efficiency and interhemispheric connectivity, were related to improved language and visual attention performances, respectively. They also found that the connectivity between the default mode network and the auditory regions was highly related to improved language, while connectivity between the salience network and visual regions was related to increasing visual attention performance. Above all, these previous studies have reflected that network topology indicators can well-predict the effectiveness of comprehensive speech therapy for patients with aphasia. Although this early study identified specific rsFC properties that predict outcomes in aphasia following treatment, the powers of these studies are usually suffered from a small sampling problem, which makes their results difficult to generalize. Thus, our study aims to extend the results of a previous study by exploring the topological properties of functional networks in patients with aphasia before comprehensive speech therapy.

Based on the dual stream model and the neuropsychological dual dissociation of structural damage and functional networks, we aim to explore changes in the whole brain functional network in aphasia after stroke. We examined the relationships between abnormal topological properties of the whole brain functional network and language behavior and cognition in aphasia after stroke. To address this issue, we used quantitative evaluations of the global and nodal characteristics of the brain functional networks and examined the relationships between these topological metrics and the aphasia battery of Chinese (ABC) (Gao, 1992), mini-mental state examination (MMSE) (Byrne et al., 2000), and the Montreal Cognitive Assessment (MoCA) (Nasreddine et al., 2005) scores.

## Materials and Methods

### Subjects

Twenty-four patients with aphasia after stroke and 19 healthy controls (HC) matched for age, sex, and years of education were recruited from the center for diagnosis and treatment of language disorders, First Affiliated Hospital of Jinan University, Guangzhou, China, from January 2016 to February 2019. All HCs and patients were right-handed and aged more than 18 years and <70 years. Inclusion criteria for the aphasia group were the following: (1) at the first onset of stroke, the lesion was confirmed to be located in the left hemisphere by cranial CT or MRI; (2) patients had received no treatment for speech recovery; (3) the duration of aphasia was 1–24 months; (3) motor aphasia diagnosis was determined by the ABC (Gao, 1992); (4) the education level of the patient was elementary school or above; and (5) they were right-handed. The exclusion criteria for all the participants were the following: (1) speech, reading, or writing impairment due to severe damage to sensory and motor organs, such as hearing, vision, articulation, and writing; (2) congenital or early childhood illnesses causing language learning difficulties and resulting in language function deficits; (3) subjects who were unconscious and unable to cooperate with examination and treatment, or who were unable to complete the scale assessment; (4) those with a history of neurological or psychiatric disorders (e.g., Alzheimer's disease, epilepsy, depression); (5) combined epilepsy, severe cardiac, hepatic, or renal dysfunction, or other serious physical illness; (6) bilateral stroke; (7) contraindications for MRI, for instance, metallic implants. The demographic and clinical information of the patients (8 women, 16 men; mean age, 51.16 ± 9.48 years) and HC (9 women, 10 men; mean age, 48.39 ± 10.66 years) is detailed in Table 1. This study was approved by the Medical Research Ethics Committee of the First Affiliated Hospital of Jinan University. Informed consent was obtained from all the subjects prior to the study.

**Table 1:** Demographic and clinical data comparisons between the patients with aphasia and healthy controls.

**Characteristic**	**Patients with aphasia (***n*** = 24)**	**Healthy controls (***n*** = 19)**	**χ^2^/***t***-value**	***p*** **-value**
Age	48.39 ± 10.66	53.70 ± 9.34	−1.71	0.094^a^
Sex (M:F)	16:8	10:9	0.48	0.490^b^
Duration (month)	5.16 ± 5.58	—	—	—
ABC (spontaneous speech)	53.08 ± 18.86	98.53 ± 1.12	−10.46	0.000*
ABC (auditory comprehension)	76.00 ± 18.24	99.95 ± 0.23	−6.43	0.000*
ABC (repetition)	74.08 ± 21.07	99.89 ± 1.12	−6.00	0.000*
ABC (naming)	54.42 ± 28.40	99.74 ± 0.57	−7.81	0.000*
language ability^c^	64.39 ± 19.20	99.53 ± 0.42	−8.96	0.000*
MMSE	14.42 ± 6.86	29.21 ± 0.98	−10.43	0.000*
MoCA	10.46 ± 6.16	28.57 ± 1.01	−14.18	0.000*

a *independent-samples t-test*;

b *chi-square test*;

c *the mean value of all sub-measures in ABC*.

* *p < 0.05, the difference is statistically significant*.

### Neuropsychological Assessments

The neuropsychological status of each patient was examined using the following scales: the ABC, the MMSE, and the MoCA (Chinese version) The ABC (Gao, 1992), which is the Chinese-standardized adaptation of the western aphasia battery, and this was applied to assess the language abilities of the patients, including spontaneous speech, auditory comprehension, repetition, and naming. The MMSE (Li et al., 2016) and MoCA (Nasreddine et al., 2005), which are widely used to systematically assess cognitive impairment. The MMSE questionnaire contains 11 indexes to measure five areas of cognitive function (attention, calculation, orientation, recall, registration, and language). The maximum MMSE score is 30, with a score <23, indicating cognitive impairment. The MoCA has eight cognitive items, including language, memory, abstraction, naming, attention, executive function, calculation, and orientation. The maximum MoCA score is 30, and a score of 26 or lower is indicative of cognitive impairment. When the number of years in education is <12, one point should be added to adjust for educational deviations (Nasreddine et al., 2005).

### MRI Data Acquisition

All MRI data were collected on a GE Discovery MR750 3.0T System equipped with an eight-channel head coil at the radiology department of the First Affiliated Hospital of Jinan University. Foam pads were used to restrict head movement, and earplugs were used to minimize scanner noise. First, conventional T1-weighted images and T2-weighted images were collected to observe brain lesions. Resting-state functional images were acquired using a gradient-recalled echo-planar imaging sequence. The sequence parameters were as follows: repetition time = 2,100 ms, echo time = 30 ms, thickness = 3.0 mm, gap = 0.6 mm, voxel size = 3.125 × 3.125 × 3.6 mm3, flip angle = 90°, matrix = 64 × 64, number of volumes = 160, number of slices = 42. Finally, high-resolution T1-weighted brain structural magnetic resonance images were obtained using a three-dimensional (3D) brain volume imaging (3D-BRAVO) sequence (repetition time = 4,500 ms, echo time = 3.22 ms, thickness = 1.0 mm, gap = 0.5 mm, field of view = 240 mm × 240 mm, flip angle = 15°, voxel size = 0.47 × 0.47 × 1.0 mm3, slices = 164).

### Image Preprocessing

The preprocessing was carried out using Data Processing and Analysis Assistant for Resting-State Brain Imaging (DPABI_V5.1) based on Statistical Parametric Mapping (SPM12) in the MATLAB 2014a platform (The MathWorks Inc., Natick, MA, US). The first 10 images of each rs-fMRI dataset were discarded, and the remaining 150 images were slice timing corrected. Then, 3D head motion correction was conducted for small head movements, which also provided a record of the head motion for image quality control. The subject whose head motion of maximum rotation was more than 3°, or maximum displacement in any direction was >3 mm, was excluded from analyses. Two imaging physicians with more than 5 years of work experience drew the 3D contours of lesions in each subject based on T1-weighted structural images, and these were used as lesion masks for normalization (see below Brett et al., 2001; Andersen et al., 2010). The T1 structural images were segmented into white matter, gray matter, and cerebrospinal fluid. For the patients, this procedure was conducted using the Clinical Toolbox (https://www.nitrc.org/projects/clinicaltbx/) supported by SPM12. Notably, as many of the subjects included in this study had large unilateral brain lesions, we used the enantiomorphic normalization with a 6-tissue parameter rather than traditional lesion-masked normalization (Rorden et al., 2012) to get a better result. For the controls, the common SPM New Segment was used. The results of segmentation were visually checked by the authors to ensure their correctness. Then, all resting-state functional images for each individual were co-registered to the T1-weighted structural images that were used for segmentation. These realigned functional images were normalized into the Montreal Neurological Institute (MNI) space with 3 × 3 × 3 resolution. The nuisance covariates (including the global signal, white matter, and cerebrospinal fluid signals, and the Friston-24-parameter model of head motions) were then removed from the functional data through linear regression to reduce the effects of confounding factors. Finally, all the standard MNI space functional images were smoothed with an 8-mm full width at half maximum Gaussian kernel, and temporal band-pass filtering (0.01–0.08 Hz) was performed.

### Functional Network Construction and Analysis

In the current study, we used the Graph Theoretical Network Analysis (GRETNA) (Wang et al., 2015) toolbox (https://www.nitrc.org/projects/gretna/) to construct the large-scale brain network. The Power-264 atlas (Power et al., 2011), a high-resolution parcellation scheme based on the results of a large meta-analysis was selected to define the nodes (i.e., functional areas) in the brain network. This atlas includes 264 putative functional areas, and its robustness for network construction has been proved by many studies (Power et al., 2013). We first extracted the time series in each node by averaging the time series of all voxels in the same nodal areas. Then, we calculated the Pearson correlation coefficient between the time series of each possible node pair. Thus, a 264 × 264 functional correlation matrix was generated for each subject. To improve the normality of the correlation coefficient, a Fisher's *r*-to-*z* transformation was applied to the correlation matrices.

A general approach to determining the available edges in a matrix is to binarize the whole matrix using a thresholding method (Achard and Bullmore, 2007). As there is no consensus in regard to the setting of functional-network-threshold to date, we employed a wide range of sparsity thresholds (0.05–0.40 with an interval of 0.05) to all the correlation matrices for the following reasons. First, we chose to threshold the functional matrix by using the network sparsity rather than the value of the matrix elements, because the latter approach will cause the extraction of different numbers of edges across subjects, which might confound group comparisons in network topology. Also, the network sparsity approach can ensure that no isolated nodes existed in any threshold point under our chosen threshold band. Second, as suggested by previous studies (Chen et al., 2019), the lower sparsity of 0.05 still ensures that the average degree is greater than the natural logarithm of the number of nodes in the Power-264 atlas, and the upper sparsity of 0.40 also does not exceed the 50% network density. Finally, as the meaning of negative correlations in the functional network is still controversial, we only included positive correlations for later network analyses.

### Functional Network Analysis

For the network analyses, we calculated both global and regional network metrics at each sparsity threshold and the area under the curve (AUC) over the sparsity range. The AUC metric provides a summarized scalar for the topological properties of brain networks that is independent of the threshold, and this is sensitive for detecting the topological alterations of brain disorders (Zhang et al., 2011; Chen et al., 2018). The global metrics included the small-world properties and network efficiency properties. Specifically, the small-world properties included five sub-measures (Watts and Strogatz, 1998): small-worldness (*sigma*), normalized clustering coefficient (*gamma*), normalized characteristic path length (*lambda*), clustering coefficient (*Cp*), and characteristic path length (*Lp*) [for the details of these sub-measures, please refer to the study of Chen et al. (2019)]. In addition, consistent with previous studies (Latora and Marchiori, 2001), we also computed the global and local network efficiencies (*E*_*glob*_/*E*_*loc*_) to overcome the shortcomings of the clustering coefficient (ignorance of indirect connections) and the shortest path length (misjudgment of isolated nodes).

In comparison to global metrics, regional metrics are better at quantitatively characterizing the nodal dynamics of brain networks (Rubinov and Sporns, 2010). In this study, we included the three node-based metrics: the nodal degree, nodal efficiency, and nodal betweenness (Achard and Bullmore, 2007; Rubinov and Sporns, 2010). The nodal degree is the original and most important measure for describing the nodal characteristics of a graph. The nodal efficiency measures the efficiency of the communication among the first neighbors of a node when it is removed, and it is very suitable for describing the local functional state in aphasia with brain injury. The nodal betweenness is defined as the fraction of all shortest paths in the network that pass through a given node, and this is suggested as a sensitive measure to detect important anatomical or functional connections (Rubinov and Sporns, 2010).

### Statistical Analysis

We used the IBM Statistical Package for the Social Sciences 20.0 software (IBM SPSS Inc., Chicago, IL, USA; https://www.ibm.com/products/spss-statistics) to compare the statistical differences in demographic and clinical data between the aphasia and the HC groups. More specifically, we used two-sample *t*-tests to detect the group differences in age, sex, duration, ABC score, MMSE score, and MoCA score, and we used chi-square tests to examine any significant sex distribution difference and duration distribution differences.

To determine whether there existed significant between-group differences in the global and the regional network metrics, we perform several two-sample *t*-tests on the AUC of each network measure (small-world metrics: *sigma, gamma, lambda, Cp, Lp*, and network efficiency metrics: *E*_*glob*_*, E*_*loc*_) between the patients with aphasia and the HC. To avoid misuse of statistical methods (e.g., when the data do not obey normal distribution), we also conducted the non-parametric permutation tests that randomly shuffle the group distribution 5,000 times to confirm the results of group comparisons. If any significant between-group differences in the network metrics were identified in the network metrics, we will further extract the AUC values of these topological properties and evaluate their relationships with the clinical data (i.e., the ABC, MMSE, and MoCA scores) in the aphasic group by using Pearson correlations. Note that as the AUC values (instead of the topological value under each threshold point) were used for statistical analyses, the potential risk of false-positive rates yielded by multiple comparisons was alleviated. Therefore, the significance level for the above analyses (the group differences in demographic and clinical data, the group differences in global network metrics, and the correlations between global network metrics and clinical data) was set at *p* < 0.05 without multiple comparison correction.

Finally, for the regional metrics (the nodal degree, nodal efficiency, and nodal betweenness), we performed two-sample *t*-tests between the patients with aphasia and the HC in each node. The Bonferroni method was used to correct the issue of multiple comparisons. In this study, given that the number of nodes was 264, the significant level for each node was set at *p* < 0.000189 (i.e., 0.05/264). However, for the exploratory purpose, we also reported the node with a more liberal threshold, which is *p* < 0.0038 (i.e., 1/264).

## Results

### Demographic and Clinical Comparisons

The demographic and clinical data from 24 patients with aphasia and the 19 HC subjects are summarized in Table 1. There were significant between-group differences in spontaneous speech, auditory comprehension, repetition, naming, language ability, MMSE, and MoCA between the patients with aphasia and healthy controls. There were no significant differences in age (*p* = 0.094) or sex (*p* = 0.49) between the patients with aphasia and healthy controls.

### Global Topological Metrics of Brain Functional Networks

In the defined threshold range (from 0.05 to 0.40, step = 0.05), we used the AUC of each global metric as an independent variable in the between-group statistical comparisons. Compared with the HC group, patients with aphasia showed significant decreased in *sigma* (*t* = −3.5065, *p* = 0.001), *gamma* (*t* = −3.637, *p* = 0.001), and *E*_*loc*_(*t* = −2.444, *p* < 0.019). No significant between-group differences were identified in the *Cp* (*t* = −1.876, *p* = 0.068), *Lp* (*t* = −0.848, *p* = 0.402), *E*_*glob*_ (*t* = 1.093, *p* = 0.281) and *Lambda* (*t* = −1.139, *p* = 0.261). In addition, we also calculated the difference of AUC values of all parameters in the threshold range (from.05 to.40, step = 0.01). Our results showed that, when choosing network sparsity, 0.01 as a step size or 0.05 as a step size did not significantly affect the pattern of the results (Supplementary Table 1). In addition to the two-sample *t*-test, we also used the non-parametric test (Mann-Whitney U-test) and permutation to compare the differences of topological properties; our results showed that the results pattern were basically the same, which indicated that the results of the *t*-test comparison between the groups in this study were reliable (Supplementary Tables 2, 3).

### Regional Topological Metrics of the Brain Functional Networks

**Figure 1:**
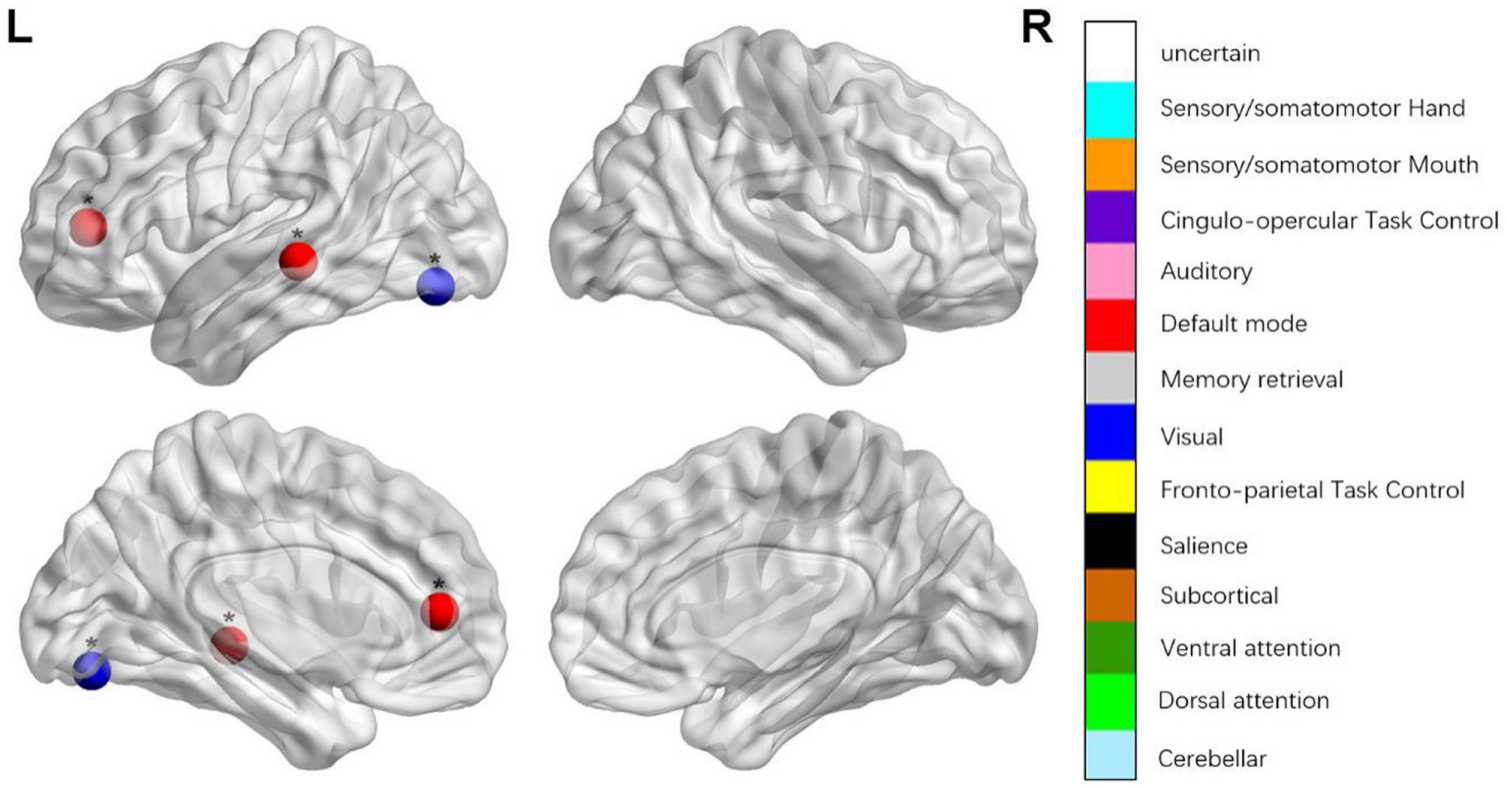
Regions were considered abnormal nodal betweenness in patients with aphasia if they exhibited significant between-group differences. *p < 0.000189, Bonferroni correction (i.e., 1/264).

**Figure 2:**
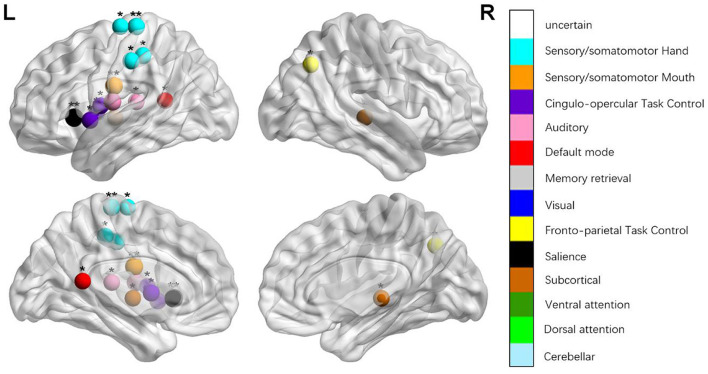
Regions were considered an abnormal nodal degree in patients with aphasia if they exhibited significant between-group differences. *p < 0.003787, Bonferroni correction (i.e., 1/264). **p < 0.000189, Bonferroni correction (i.e., 0.05/264).

**Figure 3:**
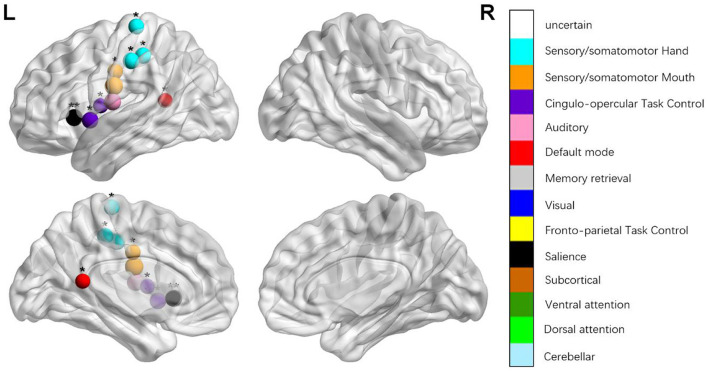
Regions were considered abnormal nodal efficiency in patients with aphasia if they exhibited significant between-group differences. *p < 0.003787, Bonferroni correction (i.e., 1/264). **p < 0.000189, Bonferroni correction (i.e., 0.05/264).

**Figure 4:**
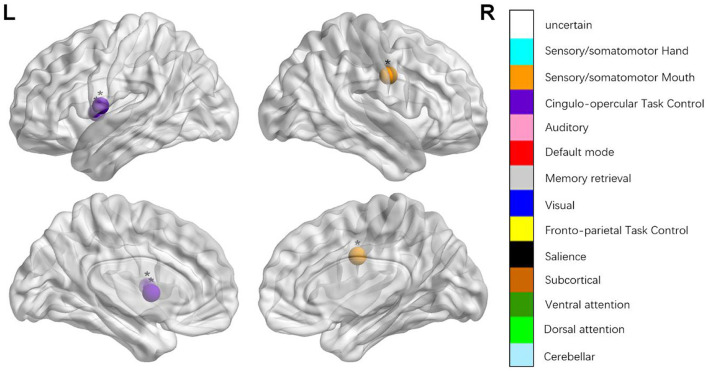
Regions were considered abnormal nodal local efficiency in patients with aphasia if they exhibited significant between-group differences. *p < 0.003787, Bonferroni correction (i.e., 1/264). **p < 0.000189, Bonferroni correction (i.e., 0.05/264).

We identified the brain regions that showed a between-group difference in the regional topological organization (^*^p < 0.000189, i.e., 1/264). Compared with the HC group, aphasia showed a decreased nodal degree in the left postcentral gyrus, central opercular cortex, and insular cortex. We also found that patients with aphasia showed decreased nodal efficiency in the left postcentral gyrus, central opercular cortex, and insular cortex (Figures 1–4 and Table 2).

**Table 2:** Between-group differences in nodal characteristics in patients with aphasia and healthy controls.

**Name**	***t***	***p***	**Power_NO**	**MNI**	**Anatomy (Harvardy)**
*BC*	−3.206	0.003	111	−11 45 8	Paracingulate grus (L)
	−3.098	0.004	118	−58 −30 −4	Posterior middle temporal gyrus (L)
	3.101	0.004	172	−33 −79 −13	Occipital fusiform gyrus (L)
*DC*	−5.537	0.000*	45	−53 −10 24	Postcentral gyrus (L)
	−4.798	0.000*	70	−55 −9 12	Central opercular cortex (L)
	−6.14	0.000*	208	−35 20 0	Insular cortex (L)
*NEg*	−5.185	0.000*	45	−53 −10 24	Postcentral gyrus (L)
	−4.347	0.000*	70	−55 −9 12	Central opercular cortex (L)
	−4.31	0.000*	208	−35 20 0	Insular cortex (L)
*NEloc*	−3.462	0.001	42	−49 −11 35	Precentral gyrus (L)
	−4.038	0.000	58	−51 8 −2	Superior temporal gyrus (L)
	3.109	0.003	91	−3 −49 13	Posterior cingulate gyrus (L)
	−3.748	0.001	44	51 −6 32	Precentral gyrus (R)
	−3.109	0.003	55	−45 0 9	Central opercular cortex (L)
	−3.595	0.001	57	−34 3 4	Insular cortex (L)

*p < 0.05, the difference is statistically significant*.

* *p < 0.000189, Bonferroni correction (i.e., 1/264); BC, betweenness centrality; DC, degree centrality; NEg, nodal efficiency; NEloc, local efficiency*.

### Brain-Behavior Correlation Analysis

Then, we used Pearson correlation to evaluate the relationships between the AUCs of sigma, gamma, and *E*_*loc*_, and the clinical variables in the aphasia group in the threshold range (from 0.05 to 0.40, step = 0.05). We found that the AUC of *E*_*loc*_ was positively correlated with the language ability (*r* = 0.5013, *p* = 0.0126), retelling (*r* = 0.4054, *p* = 0.0494), naming (*r* = 0.4054, *p* = 0.0076), listening comprehension (*r* = 0.4389, *p* = 0.0319) in patients. This pattern of correlations is not affected by the choosing network sparsity (i.e., 0.01 step or 0.05 step; see the details in Supplementary Tables 4, 5). In addition, to further ensure that the correlations are not systematically confounded by other extraneous variables, we also conducted a partial correlation between *Eloc* and clinical measures that controlled for age, sex, head motion, and the duration of the disease. The results of partial correlation confirm that Eloc was still significantly correlated with naming, listening comprehension, and language ability (Supplementary Table 6). Finally, similar correlation results were also attained by the permutation correlation procedure (Supplementary Table 7).

## Discussion

In the present study, graph-theoretical approaches were applied to assess the topological properties of brain functional networks in patients with aphasia using resting-state fMRI based on the Power-264 atlas. The results showed that patients with aphasia had a decreased sigma, gamma, and *E*_*loc*_. Moreover, patients with aphasia had decreased nodal degree and decreased nodal efficiency in the left postcentral gyrus, central opercular cortex, and insular cortex. Furthermore, *E*_*loc*_ was positively correlated with language ability, retelling, naming, and listening comprehension in patients with aphasia. Our study shows that the local efficiency of the brain network might be used as a potential indicator of basic speech function in patients with aphasia.

The dual-stream model includes a dorsal stream and a ventral stream, and these promote grammar processing and support speech production and comprehension. In contrast to the symptom–lesion view, this model envisages that the entire brain is used to process language. Following this rationale, our study considered the brain functional network as a whole and explored the changes in topological properties. In the graph theory, small-worldness is a fascinating parameter for the description of complex brain networks; it attains a much higher clustering coefficient and a similar characteristic path length and facilitates an energy-efficient balance between local specialization and global integration (Achard and Bullmore, 2007; Rubinov and Sporns, 2010). Compared with the normal control group, the patients with aphasia showed a decrease in sigma, gamma, and *E*_*loc*_. The decrease in sigma might suggest that the stroke patients with aphasia had less local specialization and global integration and that highly specialized and integrated information processing may be impaired in the brain functional network. The parameter gamma is often interpreted as an index of the strength of network segregation or specialization (Rubinov and Sporns, 2010). The decrease of gamma in stroke patients with aphasia was considered to indicate altered short-distance functional connections between adjacent regions.

The parameter *E*_*loc*_ is a metric that quantifies the capacity for transmitting information over local networks and the fault tolerance of a network (Rubinov and Sporns, 2010). Therefore, our results that patients with aphasia exhibited decreased *E*_*loc*_ might suggest that the capacity for information exchange and regional information processing have been disrupted, and it may result from the sparse or damaged functional connections. Moreover, *E*_*loc*_ was positively correlated with language ability, spontaneous expression, retelling, naming, and listening comprehension. However, there were no significant differences in the correlations between sigma, gamma, and clinical symptoms. It further illustrated that disrupted local efficiency influences cognitive impairments, information exchange and processing, and language competence in patients with aphasia. These results indicated that the topological properties of small world network had been injured in patients with aphasia, which may be caused by the impairment of language understanding and expression, memory, executive function and attention, and the local efficiency of brain networks can be a potential indicator of basic speech function in patients with aphasia. Agosta et al. (2014) found that patients with aphasia were characterized by a lower network degree, clustering coefficient, and global efficiency, as well as a longer characteristic path length and higher assortativity; and the reduced nodal degree in the inferior and ventral temporal regions and occipital cortices and the local network analysis showed that patients with aphasia were associated with a functional degradation in the “pan-modal” inferior and/or ventral temporal regions, and the “modality-specific” visual cortical origin of the ventral processing pathway. Baliki et al. (2018) used graph theory analysis to investigate the influence of anatomical and functional brain properties in response to intensive comprehensive aphasia treatment, and they found that the rsFC properties in aphasia were strongly associated with treatment outcomes instead of lesion location or size, and global efficiency and interhemispheric connectivity were related to improved language and visual attention performances, respectively. Combined with these previous studies, our results further illustrated that the network theory approach is feasible for accurate and informative mapping of brain functional networks in patients with aphasia, and that network theory might provide a more sensitive measure of brain dysfunction than those measures evaluating the properties of individual brain regions. The *E*_*loc*_ of brain network can be a potential indicator of basic speech function in patients with aphasia.

The classical framework for the study and treatment of aphasia is based on the symptom-lesion view, which considers specific behavioral and clinical symptoms in aphasia were caused by specific brain regions with particular functional specialization. Thus, the speech and language impairments that result from a stroke should be somewhat predictable (Dronkers et al., 2004). This study also explored the topological changes of each focal brain region from the local node attributes. The nodal degree is designated as the number of edges of a node that connects with the remaining nodes, evaluating the strength of a node and indicating the impact of a particular node on the overall functional network. The nodal local efficiency can be defined as a measure of the fault tolerance of a network, indicating how well each subgraph exchanges information when the node is eliminated (Sporns, 2011). Compared with the HCs, patients with aphasia showed changed nodal degree and nodal local efficiency in the left insular cortex, postcentral gyrus, and central opercular cortex.

The insular cortex is an important part of the language center, and it is involved in the formation and expression of language, as well as explicit and implicit learning of grammar. It is closely associated with language comprehension (Wernicke area), language repetition (superior gyrus), language production (Broca area), and other language-related areas (Jakab et al., 2012). Patients with aphasia showed lower nodal degree and lower nodal local efficiency in the insular cortex, which may be the reason for their lower language fluency, language comprehension, and language repetition. Peter et al. conducted a positron emission tomography study and found that several regions, including the left anterior insula/prefrontal lobe, showed low metabolism in aphasia, which indicated that the decreased language fluency was due to the defect of a motor pronunciation plan (apraxia of speech) caused by the defects of the insula (Nestor et al., 2003). Patients with aphasia also showed changed nodal degree and nodal local efficiency in the left postcentral gyrus, central opercular cortex, which is the part of the dorsal stream (Hickok and Poeppel, 2007; Fridriksson et al., 2016). The dorsal stream (involving the fronto-parietal regions, including the pars opercularis, pars triangularis, pre- and post-central regions, and portions of the parietal lobe (Fridriksson et al., 2016) processes auditory-to-articulation information, including the feedback necessary for predicting the spoken output and online error detection and modification of speech output, and it provides *ad hoc* auditory and proprioceptive feedback that is crucial for producing fluent speech (Hickok and Poeppel, 2007; Fridriksson et al., 2016). The decreased nodal degree and nodal efficiency in the left postcentral gyrus and central opercular cortex region, indicate the damage to the dorsal stream and the impaired motor speech production functions, leading to the production of non-fluent speech. We maintain that the decreased nodal properties of local brain regions can further explain the motor speech and comprehension impairment.

There are some limitations to our study. First, the course of disease of the subjects varied greatly, and future studies should select more rigorous samples and have a larger sample size to verify the results of this study. Second, to further explore the link between network attributes and the mechanisms of aphasia, future studies should include longitudinal comparisons of treatments. Third, only one network template (Power-264) was calculated in this study, and future studies should compare whether the results of different network templates are different and whether there are more closely related clinical indicators in the process and network template selection.

## Conclusion

In our study, graph-based theoretical approaches were applied to assessing the altered topological properties of brain functional networks in patients with aphasia using resting-state fMRI. The results show that patients with aphasia had decreased sigma, gamma, and *E*_*loc*_. Notably, the *E*_*loc*_were positively correlated with language ability, retelling, naming, and listening comprehension in patients with aphasia. Moreover, patients with aphasia had decreased nodal degree and nodal efficiency in the left postcentral gyrus, central opercular cortex, and insular cortex. We argue that the local efficiency of the brain network might be used as a potential indicator of basic speech function in patients with aphasia.

## Data Availability Statement

The raw data supporting the conclusions of this article will be made available by the authors, without undue reservation.

## Ethics Statement

The studies involving human participants were reviewed and approved by the Medical Research Ethics Committee of The First Affiliated Hospital of Jinan University. The patients/participants provided their written informed consent to participate in this study.

## Author Contributions

XC contributed to the study design, execution, methodology, analysis, manuscript drafting, writing—review, and editing. LC and SZ contributed to methodology, software analysis, manuscript drafting and writing, review, and editing. XC, HW, and YD contributed to the execution of the study and reviewed the article. ZC and RH were involved in the conceptualization, funding acquisition, project administration, resources, and supervision. All authors read and approved the final manuscript.

## Funding

This work was co-funded by the Medical Science and Technology Research Fund of Guangdong Province, China, Grant Nos. A2019407, A2021113 and the Project of Administration of Traditional Chinese Medicine of Guangdong Province of China, Grant No. 20201080.

## Conflict of Interest

The authors declare that the research was conducted in the absence of any commercial or financial relationships that could be construed as a potential conflict of interest.

## Publisher's Note

All claims expressed in this article are solely those of the authors and do not necessarily represent those of their affiliated organizations, or those of the publisher, the editors and the reviewers. Any product that may be evaluated in this article, or claim that may be made by its manufacturer, is not guaranteed or endorsed by the publisher.
