# Supplementary material for: Disrupted Brain Connectivity Networks in Aphasia Revealed by Resting-State fMRI
Source: Front Aging Neurosci. 2021 Oct 20;13:666301. doi: 10.3389/fnagi.2021.666301 (PMC8565294; doi:10.3389/fnagi.2021.666301)
Supplement: Supplementary file 1 [file Data_Sheet_1.docx]

**Supplemental material**

Table 1 The difference of AUC values of all parameters in the threshold range (from 0.05 to 0.40, step = 0.01).

|  |  |  | **NoGlobal** | | **Global** | |
| --- | --- | --- | --- | --- | --- | --- |
| **Step** | **Name** | **Parameter** | ***t*** | ***p*** | ***t*** | ***p*** |
| 0.05 | Network Efficiency | Eg | -1.1183 | 0.2699 | 1.0933 | 0.2807 |
|  |  | Eloc | **-3.1941** | **0.0027** | **-2.4443** | **0.0189** |
|  | Small World | Cp | -1.0611 | 0.2948 | -1.8761 | 0.0678 |
|  |  | Gamma | -1.6118 | 0.1147 | **-3.6371** | **0.0008** |
|  |  | Lambda | 0.0389 | 0.9692 | -1.1388 | 0.2614 |
|  |  | Lp | 1.3830 | 0.1741 | -0.8477 | 0.4015 |
|  |  | Sigma | -1.5250 | 0.1349 | **-3.5065** | **0.0011** |
| 0.01 | Network Efficiency | Eg | -1.0774 | 0.2876 | 1.2009 | 0.2367 |
|  |  | Eloc | **-3.2440** | **0.0023** | **-2.4162** | **0.0202** |
|  | Small World | Cp | -1.0848 | 0.2843 | -1.8754 | 0.0679 |
|  |  | Gamma | -1.6558 | 0.1054 | **-3.5557** | **0.0010** |
|  |  | Lambda | -0.0317 | 0.9749 | -1.2871 | 0.2053 |
|  |  | Lp | 1.2742 | 0.2098 | -1.0739 | 0.2892 |
|  |  | Sigma | -1.5657 | 0.1251 | **-3.4418** | **0.0013** |

We also calculated the difference of AUC values of all parameters in the threshold range (from 0.05 to 0.40, step = 0.01). Our results showed that when choosing network sparsity, 0.01 as a step size or 0.05 as a step size did not significantly affect the pattern of the results.

Table 2 The non-parametric test (Mann-Whitney U test) to compare the differences of topological properties

|  |  |  | **NoGlobal** | | **Global** | |
| --- | --- | --- | --- | --- | --- | --- |
| **Step** | **Name** | **Parameter** | ***Z*** | ***p*** | ***Z*** | ***p*** |
| 0.05 | Network Efficiency | Eg | -1.0394 | 0.2986 | 1.5285 | 0.1264 |
|  |  | Eloc | **-3.0203** | **0.0025** | **-2.6045** | **0.0092** |
|  | Small World | Cp | -1.2106 | 0.2261 | **-2.1888** | **0.0286** |
|  |  | Gamma | -1.4796 | 0.1390 | **-3.4360** | **0.0006** |
|  |  | Lambda | -0.4769 | 0.6334 | -1.8953 | 0.0580 |
|  |  | Lp | 1.2595 | 0.2079 | -1.4062 | 0.1597 |
|  |  | Sigma | -1.3328 | 0.1826 | **-3.1915** | **0.0014** |
| 0.01 | Network Efficiency | Eg | -0.8926 | 0.3721 | 1.5774 | 0.1147 |
|  |  | Eloc | **-2.9958** | **0.0027** | **-2.5312** | **0.0114** |
|  | Small World | Cp | -1.2106 | 0.2261 | **-2.2132** | **0.0269** |
|  |  | Gamma | -1.4307 | 0.1525 | **-3.5339** | **0.0004** |
|  |  | Lambda | -0.5258 | 0.5990 | **-2.1154** | **0.0344** |
|  |  | Lp | 1.1127 | 0.2658 | -1.5285 | 0.1264 |
|  |  | Sigma | -1.3818 | 0.1670 | **-3.1181** | **0.0018** |

Table 3 The permutation to compare the differences of topological properties.

|  |  |  | **NoGlobal** | | **Global** | |
| --- | --- | --- | --- | --- | --- | --- |
| **Step** | **Name** | **Parameter** | ***perT*** | ***perD*** | ***perT*** | ***perD*** |
| 0.05 | Network Efficiency | Eg | 0.2774 | 0.2708 | 0.2830 | 0.2742 |
|  |  | Eloc | **0.0036** | **0.0014** | **0.0206** | **0.0184** |
|  | Small World | Cp | 0.3006 | 0.3050 | 0.0644 | 0.0626 |
|  |  | Gamma | 0.1190 | 0.1142 | **0.0004** | **0.0016** |
|  |  | Lambda | 0.9666 | 0.9674 | 0.2616 | 0.2542 |
|  |  | Lp | 0.1854 | 0.1824 | 0.4066 | 0.4042 |
|  |  | Sigma | 0.1378 | 0.1324 | **0.0008** | **0.0014** |
| 0.01 | Network Efficiency | Eg | 0.2780 | 0.2918 | 0.2378 | 0.2326 |
|  |  | Eloc | **0.0018** | **0.0020** | **0.0212** | **0.0208** |
|  | Small World | Cp | 0.2926 | 0.2754 | 0.0656 | 0.0616 |
|  |  | Gamma | 0.1092 | 0.1040 | **0.0004** | **0.0008** |
|  |  | Lambda | 0.9732 | 0.9754 | 0.2112 | 0.2016 |
|  |  | Lp | 0.2100 | 0.2094 | 0.2876 | 0.2902 |
|  |  | Sigma | 0.1196 | 0.1300 | **0.0020** | **0.0016** |

Table 2 & 3 We used the non-parametric test (Mann-Whitney U test) and permutation to compare the differences of topological properties, our results showed that the results were basically the same, which indicated that the results of the t-test comparison between the groups in this study were reliable.

Table 4 The Pearson correlation to evaluated the relationships between the AUC of sigma, gamma and Eloc and the clinical variables in the aphasia group in the threshold range (from 0.05 to 0.40, step = 0.05).

| **R value table of clinical indicators and brain network parameters: Step = 0.05** | | | | | | | |
| --- | --- | --- | --- | --- | --- | --- | --- |
| **Parameter** | **Se** | **Re** | **Na** | **Co** | **LA** | **MMSE** | **MoCa** |
| **No Global** |  |  |  |  |  |  |  |
| Eg | -0.0192 | 0.0802 | -0.0513 | 0.1496 | 0.0301 | 0.1385 | 0.1764 |
| Eloc | 0.3890 | **0.4054** | **0.5311** | **0.4389** | **0.5013** | 0.2401 | 0.2801 |
| Cp | 0.2275 | 0.1745 | 0.3370 | 0.1377 | 0.2602 | 0.0329 | 0.0445 |
| Gamma | 0.2103 | 0.3001 | 0.2670 | 0.3458 | 0.3084 | 0.3504 | 0.2670 |
| Lambda | 0.0521 | 0.0380 | 0.1519 | 0.1620 | 0.1178 | 0.0515 | 0.0929 |
| Lp | -0.0092 | -0.1204 | -0.0069 | -0.1281 | -0.0641 | -0.1382 | -0.1115 |
| Sigma | 0.1905 | 0.2770 | 0.2263 | 0.3112 | 0.2743 | 0.3308 | 0.2508 |
| **Global** |  |  |  |  |  |  |  |
| Eg | -0.2082 | -0.2740 | -0.2910 | -0.1911 | -0.2744 | -0.1766 | -0.1903 |
| Eloc | 0.3802 | 0.3856 | **0.4500** | 0.3367 | **0.4397** | 0.2711 | 0.3056 |
| Cp | 0.3199 | 0.3514 | 0.4025 | 0.2759 | 0.3838 | 0.2413 | 0.2667 |
| Gamma | 0.3865 | 0.2501 | 0.3900 | 0.3965 | 0.3998 | 0.2999 | 0.3322 |
| Lambda | 0.2579 | 0.3116 | 0.3356 | 0.2492 | 0.3267 | 0.2305 | 0.2374 |
| Lp | 0.1808 | 0.2604 | 0.2735 | 0.1737 | 0.2535 | 0.1645 | 0.1646 |
| Sigma | 0.3437 | 0.1962 | 0.3370 | 0.3648 | 0.3483 | 0.2596 | 0.2844 |
| **P-value table of clinical indicators and brain network parameters** | | | | | | | |
| **Parameter** | **Se** | **Re** | **Na** | **Co** | **LA** | **MMSE** | **MoCa** |
| **No Global** |  |  |  |  |  |  |  |
| Eg | 0.9291 | 0.7095 | 0.8117 | 0.4855 | 0.8889 | 0.5188 | 0.4096 |
| Eloc | **0.0603** | **0.0494** | **0.0076** | **0.0319** | **0.0126** | 0.2585 | 0.1849 |
| Cp | 0.2849 | 0.4147 | 0.1073 | 0.5210 | 0.2195 | 0.8787 | 0.8363 |
| Gamma | 0.3239 | 0.1542 | 0.2073 | **0.0979** | 0.1426 | **0.0933** | 0.2072 |
| Lambda | 0.8091 | 0.8601 | 0.4785 | 0.4496 | 0.5836 | 0.8111 | 0.6659 |
| Lp | 0.9661 | 0.5754 | 0.9744 | 0.5509 | 0.7660 | 0.5194 | 0.6039 |
| Sigma | 0.3725 | 0.1900 | 0.2876 | 0.1389 | 0.1946 | 0.1144 | 0.2371 |
| **Global** |  |  |  |  |  |  |  |
| Eg | 0.3290 | 0.1951 | 0.1677 | 0.3712 | 0.1944 | 0.4090 | 0.3731 |
| Eloc | **0.0669** | **0.0627** | **0.0274** | 0.1076 | **0.0315** | 0.2000 | 0.1465 |
| Cp | 0.1275 | **0.0923** | 0.0512 | 0.1920 | 0.0641 | 0.2559 | 0.2078 |
| Gamma | **0.0621** | 0.2386 | **0.0595** | **0.0551** | **0.0529** | 0.1544 | 0.1127 |
| Lambda | 0.2237 | 0.1383 | 0.1089 | 0.2403 | 0.1192 | 0.2785 | 0.2640 |
| Lp | 0.3978 | 0.2191 | 0.1959 | 0.4169 | 0.2320 | 0.4425 | 0.4422 |
| Sigma | 0.1001 | 0.3582 | 0.1073 | **0.0797** | **0.0953** | 0.2206 | 0.1781 |

Table 4 The Pearson correlation to evaluated the relationships between the AUC of sigma, gamma and Eloc and the clinical variables in the aphasia group in the threshold range (from 0.05 to 0.40, step = 0.01).

| **R value table of clinical indicators and brain network parameters: Step = 0.01** | | | | | | | |
| --- | --- | --- | --- | --- | --- | --- | --- |
| **Parameter** | **Se** | **Re** | **Na** | **Co** | **LA** | **MMSE** | **MoCa** |
| **No Global** |  |  |  |  |  |  |  |
| Eg | -0.0181 | 0.0815 | -0.0551 | 0.1513 | 0.0297 | 0.1361 | 0.1803 |
| Eloc | 0.3922 | **0.4135** | **0.5396** | **0.4431** | **0.5083** | 0.2505 | 0.2770 |
| Cp | 0.2272 | 0.1742 | 0.3410 | 0.1389 | 0.2618 | 0.0379 | 0.0426 |
| Gamma | 0.2091 | 0.3032 | 0.2686 | 0.3494 | 0.3103 | 0.3567 | 0.2688 |
| Lambda | 0.0510 | 0.0562 | 0.1736 | 0.1714 | 0.1323 | 0.0497 | 0.0678 |
| Lp | 0.0050 | -0.0972 | 0.0252 | -0.1268 | -0.0424 | -0.1306 | -0.1338 |
| Sigma | 0.1905 | 0.2788 | 0.2267 | 0.3135 | 0.2754 | 0.3370 | 0.2552 |
| **Global** |  |  |  |  |  |  |  |
| Eg | -0.2185 | -0.2835 | -0.2927 | -0.2004 | -0.2822 | -0.1832 | -0.1981 |
| Eloc | 0.3802 | 0.3848 | **0.4490** | 0.3401 | **0.4400** | 0.2692 | 0.3054 |
| Cp | 0.3182 | 0.3481 | 0.4005 | 0.2745 | 0.3815 | 0.2381 | 0.2647 |
| Gamma | 0.3987 | 0.2602 | **0.4126** | **0.4074** | **0.4164** | 0.3211 | 0.3505 |
| Lambda | 0.2627 | 0.3203 | 0.3344 | 0.2589 | 0.3319 | 0.2321 | 0.2428 |
| Lp | 0.2000 | 0.2767 | 0.2802 | 0.1908 | 0.2689 | 0.1760 | 0.1794 |
| Sigma | 0.3607 | 0.2090 | 0.3671 | 0.3779 | 0.3702 | 0.2847 | 0.3067 |
| **P-value table of clinical indicators and brain network parameters** | | | | | | | |
| **Parameter** | **Se** | **Re** | **Na** | **Co** | **LA** | **MMSE** | **MoCa** |
| **No Global** |  |  |  |  |  |  |  |
| Eg | 0.9331 | 0.7050 | 0.7980 | 0.4805 | 0.8905 | 0.5259 | 0.3992 |
| Eloc | **0.0580** | **0.0446** | **0.0065** | **0.0301** | **0.0112** | 0.2377 | 0.1901 |
| Cp | 0.2858 | 0.4156 | 0.1029 | 0.5175 | 0.2166 | 0.8603 | 0.8434 |
| Gamma | 0.3268 | 0.1498 | 0.2044 | **0.0942** | 0.1400 | 0.0871 | 0.2041 |
| Lambda | 0.8130 | 0.7943 | 0.4173 | 0.4232 | 0.5378 | 0.8178 | 0.7530 |
| Lp | 0.9815 | 0.6514 | 0.9070 | 0.5549 | 0.8439 | 0.5430 | 0.5332 |
| Sigma | 0.3727 | 0.1871 | 0.2867 | 0.1358 | 0.1927 | 0.1073 | 0.2288 |
| **Global** |  |  |  |  |  |  |  |
| Eg | 0.3050 | 0.1795 | 0.1652 | 0.3478 | 0.1815 | 0.3916 | 0.3535 |
| Eloc | **0.0669** | **0.0634** | **0.0277** | 0.1039 | **0.0314** | 0.2033 | 0.1467 |
| Cp | 0.1297 | **0.0955** | **0.0525** | 0.1942 | **0.0658** | 0.2626 | 0.2114 |
| Gamma | **0.0536** | 0.2195 | **0.0451** | **0.0481** | **0.0429** | 0.1261 | **0.0931** |
| Lambda | 0.2150 | 0.1270 | 0.1102 | 0.2219 | 0.1131 | 0.2752 | 0.2530 |
| Lp | 0.3488 | 0.1905 | 0.1848 | 0.3719 | 0.2039 | 0.4107 | 0.4016 |
| Sigma | **0.0833** | 0.3271 | **0.0776** | **0.0687** | **0.0750** | 0.1775 | 0.1449 |

Table 4 & 5 This pattern of correlations dose not affected by the choosing network sparsity (i.e., 0.01 step or 0.05 step)

Table 6 Partial correlation between Eloc and clinical variables

|  | | | | | | | | | |
| --- | --- | --- | --- | --- | --- | --- | --- | --- | --- |
| **Global** | **Step** |  | **Se** | **Re** | **Na** | **Co** | **LA** | **MMSE** | **MoCa** |
| No | 0.05 | *r* | 0.3751 | 0.3348 | **0.5800** | **0.5256** | **0.5189** | **0.3865** | 0.3126 |
|  |  | *p* | 0.1032 | 0.1491 | **0.0073** | **0.0173** | **0.0191** | **0.0923** | 0.1797 |
| No | 0.01 | *r* | 0.3778 | 0.3398 | **0.5836** | **0.5272** | **0.5225** | **0.4005** | 0.3157 |
|  |  | *p* | 0.1005 | 0.1427 | **0.0069** | **0.0169** | **0.0181** | **0.0801** | 0.1751 |
| Yes | 0.05 | *r* | 0.3591 | 0.2809 | **0.4117** | 0.3591 | **0.3995** | **0.4130** | 0.3634 |
|  |  | *p* | 0.1199 | 0.2303 | **0.0713** | 0.1200 | **0.0810** | **0.0703** | 0.1152 |
| Yes | 0.01 | *r* | 0.3603 | 0.2835 | **0.4126** | 0.3645 | **0.4020** | **0.4077** | 0.3581 |
|  |  | *p* | 0.1186 | 0.2258 | **0.0707** | 0.1141 | **0.0789** | **0.0744** | 0.1211 |

*E_loc_* was significantly associated with naming (NA), listening comprehension (CO), and mean aphasia (LA) in the absence of whole-brain preprocessing, although covariate removal reduced the significance of some of these results.

Table 7 The permutation to evaluated the relationships between the AUC of *E_loc_* and the clinical variables in the aphasia group

|  | | | | | | | | | |
| --- | --- | --- | --- | --- | --- | --- | --- | --- | --- |
| **Global** | **Step** |  | **Se** | **Re** | **Na** | **Co** | **LA** | **MMSE** | **MoCa** |
| No | 0.05 |  | **0.055** | **0.05** | **0.0076** | **0.0306** | **0.0106** | 0.2494 | 0.1762 |
| No | 0.01 |  | **0.0574** | **0.041** | **0.0036** | **0.0308** | **0.0094** | 0.236 | 0.1906 |
| Yes | 0.05 |  | **0.0668** | **0.0654** | **0.0232** | **0.0992** | **0.0324** | 0.191 | 0.1374 |
| Yes | 0.01 |  | **0.0672** | **0.0622** | **0.0288** | 0.1064 | **0.0338** | 0.2082 | 0.1528 |

The significance measured by the permutation correlation analysis is almost identical to that obtained by the original R value.
